# Supplementary material for: Structural Properties of Casein Micelles with Adjusted Micellar Calcium Phosphate Content
Source: Foods. 2024 Jan 19;13(2):322. doi: 10.3390/foods13020322 (PMC10815582; doi:10.3390/foods13020322)
Supplement: Supplementary file 1 [file foods-13-00322-s001.zip › foods-2823494-supplementary.pdf]

Supplementary data

Table S1. Changes of structural features of proteins in Amide I region in MCP-adjusted skim milk samples as determined by Fourier transform Infrared Spectroscopy. MCP-adjusted skim milk samples containing from 7% (MCP_7) to 129% (MCP_{129}) of MCP relative to that of the control were obtained by either acidification or alkalisation followed by exhaustive dialysis against bulk milk. The spectra were obtained after the background adjustment.

**Band Assessment**	**Band Frequency (cm^{-1})**	**MCP_7**	**MCP_{26}**	**MCP_{31}**	**MCP_{42}**	**MCP_{58}**	**MCP_{67}**	**MCP_{100}**	**MCP_{113}**	**MCP_{129}**
Side chain	1608-1611	$12.9±0.86^{d}$	$16.3±0.10^{c}$	$12.7±0.49^{de}$	$12.2±1.24^{de}$	$13.2±0.60^{cd}$	$9.9±0.56^{de}$	$9.5±0.15^{e}$	$23.1±1.76^{b}$	$27.3±0.35^{a}$
β-sheet	1620-1631	$34.6±5.03^{abc}$	$35.3±0.18^{ab}$	$38.7±3.70^{ab}$	$41.7±3.03^{a}$	$32.2±3.32^{abc}$	$32.3±1.63^{abc}$	$30.1±2.5^{bcd}$	$19.2±1.10^{d}$	$23.7±0.35^{cd}$
Random coil	1640-1649	$13.2±0.84^{ab}$	$16.4±0.13^{ab}$	$13.7±0.95^{ab}$	$17.4±6.74^{ab}$	$21.5±5.55^{ab}$	$22.2±3.54^{ab}$	$23.5±6.32^{a}$	$16.2±3.72^{ab}$	$7.5±0.15^{b}$
α-helix	1658-1666	$5.5±0.60^{a}$	$2.8±0.12^{ab}$	$3.7±0.81^{ab}$	$1.9±0.01^{b}$	$2.7±1.66^{ab}$	$2.7±0.86^{ab}$	$2.7±1.29^{ab}$	$3.5±0.97^{ab}$	$5.1±0.24^{a}$
β-turn	1668-1681	$23.8±1.89^{abc}$	$21.6±0.34^{bc}$	$23.7±2.31^{abc}$	$17.8±1.61^{c}$	$22.4±2.70^{abc}$	$24.9±0.57^{abc}$	$25.3±0.53^{abc}$	$29.9±4.08^{a}$	$29.4±0.51^{ab}$
Aggregated β-sheet	1689-1694	$10.0±0.81^{a}$	$7.6±0.17^{a}$	$7.6±1.03^{a}$	$8.8±0.85^{a}$	$7.7±0.44^{a}$	$8.0±1.10^{a}$	$8.7±2.13^{a}$	$8.1±0.06^{a}$	$7.1±0.10^{a}$

The subscripts indicate proportion of retained MCP relative to that of the control; [2]The small letters show significant differences within the columns ($p < 0.05$); the results are expressed as means ± standard deviation.

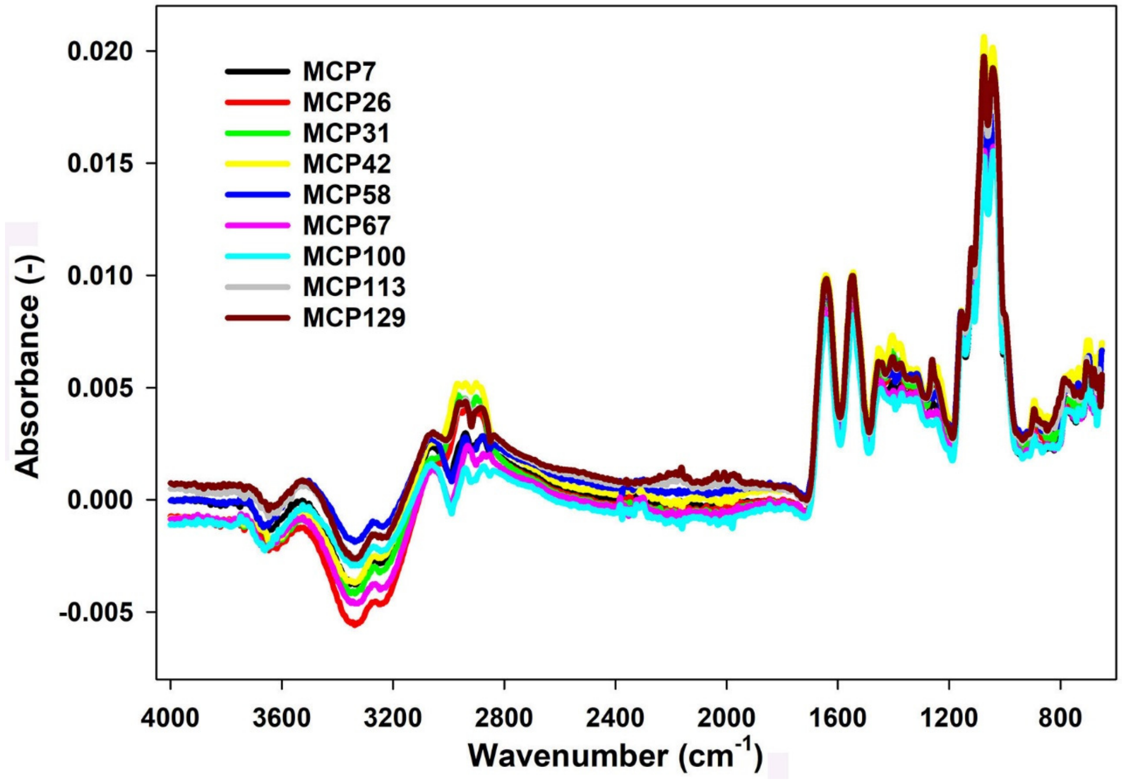

Figure S1. The original FTIR spectra (4000-650 cm^{-1}) of MCP adjusted skim milk varied containing from 7% (MCP_7) to 129% (MCP_{129}) of MCP relative to that of the control achieved by either acidification or alkalisation followed by exhaustive dialysis against bulk milk.
